# Large femoral bone loss after hip revision using the  uncemented proximally porous-coated Bi-Metric prosthesis: 22 hips followed for a mean of 6 years

**DOI:** 10.1080/17453670902804802

**Published:** 2009-02-01

**Authors:** Per Y Adolphson, Mats OF Salemyr, Olof G Sköldenberg, Henrik SG Bodén

**Affiliations:** Department of Clinical Sciences at Danderyd Hospital, Karolinska InstitutetStockholmSweden

## Abstract

**Background and purpose** Periprosthetic bone loss after uncemented femoral hip revision is a matter of concern. We have used a proximally porous- and hydroxyapatite-coated prosthesis (Bi-Metric) in revision since 1989 and now we report the bone changes. This prosthesis is intended to distribute the forces more evenly and to avoid proximal femoral unloading.

**Methods** 22 patients were unilaterally reoperated because of aseptic loosening. Only patients with a healthy contralateral hip were included. Mean age at revision was 69 (55–80) years. Bone defects were graded by Gustilo-Pasternak and Endo-Klinik classifications. Clinical assessment was performed with Harris hip score. We used radiographs and dual-energy X-ray absorptiometry to evaluate migration, femoral remodeling, and bone mineral density after 72 (30–158) months.

**Results** The mean Harris hip score was 74 (30–100) points at follow-up. Mild thigh discomfort was present in 1 patient and moderate thigh pain in 3 patients. There was no loosening or subsidence. Osteolysis seen at revision had diminished in 19 of the 22 hips at follow-up. We noted a large reduction in bone mineral density. It was most pronounced in Gruen regions 1, 2, 6, and 7.

**Interpretation** Revision with this stem is a reliable procedure; however, we noted a large degree of proximal bone loss that could lead to later mechanical complications or fractures.

## Introduction

Uncemented hip revision after aseptic loosening has been promising in the short to medium time interval (Böhm and Bischel 2001, Moreland and Moreno 2001, Trikha et al. 2005, Salemyr et al. 2008) and several authors have reported good results in the long-term perspective as well (Wagner 1987, Paprosky et al. 1999, Raman et al. 2005, Reikerås and Gunderson 2006). Most stems used in these studies are extensively coated or distally anchored. A disadvantage of these stems is the risk of stress-shielding, reducing the proximal bone stock and transferring excessive load distally. We have found substantial radiographic signs of bone recovery after secondary uncemented total hip arthroplasty with the proximally porous and hydroxyapatite- (HA-) coated Bi-Metric femoral stem (Salemyr et al. 2008). The present study was undertaken to investigate the bone changes with DEXA after the same procedure. We now report the bone changes after an average follow-up time of 6 years and compare the changes with earlier results from primary uncemented arthroplasty using the same prosthesis.

## Patients and methods

All 60 patients who were reoperated at our department with the proximally porous and HA-coated tapered Bi-Metric stem between 1989 and 2002 due to aseptic femoral loosening were reviewed retrospectively. At follow-up 8 patients had died, 7 of whom had still had their stem in place. Only patients with a healthy contralateral hip were included (28 patients). 6 patients who got their index prosthesis because of a fracture (4 hips), inflammatory arthritis (1 hip), or dysplasia (1 hip) were excluded due to their different bone metabolism. 1 patient only attended the radiographic part of the study. This left 22 patients (22 hips) for the study.

**Figure 1.**
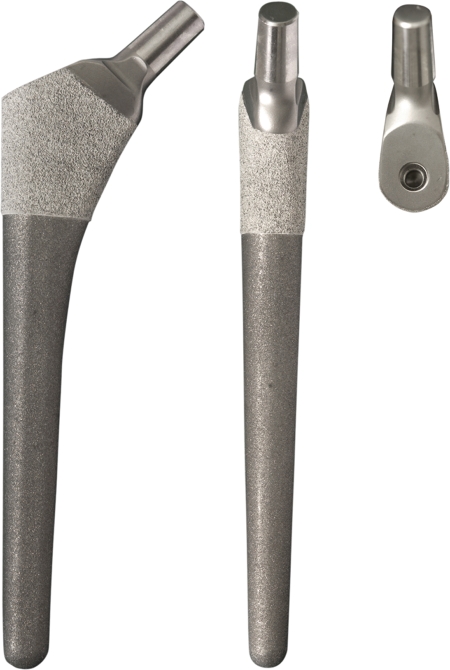
The proximally hydroxyapatite-coated Bi-Metric modular femoral stem. The prosthesis is tapered in 3 dimensions.

Mean age at revision was 69 (55–80) years and the mean follow-up time was 6 (2.5–13) years. The index diagnosis was primary osteoarthritis in all patients. 16 patients had had only 1 hip arthroplasty in the same hip before the revision, 4 patients had had 2 previous arthroplasties, and 2 patients had undergone 3 earlier arthroplasties. Mean time between the primary hip arthroplasty and our revision was 6 (1–13) years. The patients were reoperated with an uncemented prosthesis: the Bi-Metric femoral stem (Biomet Inc., Warsaw, IN) (Figure 1). It is a collarless, tapered stem (3º) made of titanium alloy, where the proximal 30% of the stem has a porous-coated (100–200 µm) surface with a plasma-sprayed HA layer (thickness 40–70 µm, crystallinity 50–70%, purity > 95%). The distal 70% has a textured surface with a roughness of 6.9 µm. It has incremental sizing from 7–19 mm in diameter, has a proportional increase in length from 115 to 175 mm, and has a modular head of cobalt chrome. All patients were reoperated through a posterior approach. 19 of the revised stems were cemented. Bone grafting around the proximal part of the stem was simultaneously carried out with small amounts of autologous bone (11 hips) and homologous bone (2 hips). 11 patients underwent a simultaneous cup exchange at revision, and 2 later cup revisions were also done. The patients received either a cemented polyethylene cup (8 patients) or an uncemented hydroxyapatite-coated cup with a polyethylene liner (5 patients). The patients were mobilized on the day after the operation under supervision of a physiotherapist. Postoperative weight bearing was individualized according to the surgeon's preference. Some patients were recommended protected weight bearing for up to 3 months.

### Clinical evaluation

The patients were evaluated clinically, with interview and examination performed by one author (MS) who had not been involved in the operations. He categorized the patients' functional status according to Charnley's classification (1972). 13 patients had unilateral disease (group A), no patient had bilateral disease (group B) by virtue of selection criterion, and 9 patients were classified as group C, i.e. they had disabilities other than the hip, which interfered with their functional or locomotor capacity. Clinical outcome was assessed with Harris hip score (HHS). The patients were also asked if they suffered or had suffered mid-thigh pain. The pain was categorized as being mild, moderate, or severe.

### Radiographic evaluation

Standardized anterioposterior and lateral radiographs after the index operation were compared with radiographs before and immediately after the revision and with radiographs taken at the time of survey. All radiographs were reviewed by MS. He recorded bone deficiencies prior to revision, stem migration, bone remodeling, and periprosthetic femoral bone loss. Bone defects prior to revision were classified according to the Gustilo and Pasternak (1988) and Endo-Klinik (Engelbrecht and Siegel 1989) classifications (Table 1). Bone defects were mostly of type II in both classifications and no type IV defect was observed. Migration was recorded as subsidence and change in varus-valgus orientation. Subsidence was determined by comparing serial radiographs, measuring the difference in distance between a horizontal line drawn at the inferior margin of the HA coating and the tip of the greater trochanter and/or between the horizontal line and the most medial point of the lesser trochanter. Varus-valgus orientation was evaluated by measuring the angle between the vertical axis of the femur and the vertical axis of the stem. Stem subsidence of > 4 mm (Malchau et al. 1995) or change in varus-valgus orientation of > 2º (Aldinger et al. 2003) was considered as definite migration.

**Table 1.** Femoral bone defects at revision (22 hips)

Type	Gustilo and Pasternak	Endo-Klinik
I	6	1
II	16	17
III	0	4
IV	0	0

The stability of the femoral component was assessed by the criteria of Engh et al. (1990). A component was defined as having fixation by bone ingrowth if there was no subsidence and minimal or no formation of a radio-opaque line along the porous-coated portion of the implant. The presence of radiolucencies, shown as linear and focal osteolysis, was assessed in 7 regions in the coronal plane using the regions described by Gruen. Progression or regression of osteolysis was recorded. Formation of areas with new bone resorption or osteolysis was also noted. New endosteal bone bridges (spot welds), distal cortical hypertrophy, and distal endosteal bone bridging (pedestal formation) were assessed as described by Engh et al. (1987). Signs of stress-shielding were recorded as calcar resorption, calcar round-off (a less intense phenomenon of the same nature), or cortical hypertrophy. Heterotopic ossification was graded according to Brooker et al. (1973).

### Evaluation of bone mineral

We measured the bone mineral density (BMD) of the periprosthetic femur in the coronal plane with a dual-energy X-ray absorptiometer, DEXA (DPX-L; Lunar Co., Madison, WI). During scanning, the patient was placed supine with standard knee and foot supports, and with the femur in neutral rotation. The scanner was equipped with software for femoral periprosthetic bone mineral measurement. The operated hip was scanned and BMD values in 7 Gruen regions of interest were analyzed. A simulated prosthesis mask was automatically superimposed on the unoperated side and adjusted, with the lesser trochanter as reference, to ensure that the same regions were scanned on both hips. The values were expressed as areal BMD, in g/cm^2^. The median change in BMD was expressed as a percentage of that on the unoperated side for the 7 regions.

Informed consent was obtained from each patient in the study. According to the ethics board at Karolinska Institute, no permission was required for this study (04-453/3). The investigation was approved by the coSORTtee for protection against radiation at Danderyd Hospital (2003-3).

### Precision

To estimate the precision error of the DEXA method, we had earlier made double measurements in 10 patients, with complete repositioning of the patient and the scanner. We found a precision error of 1–4% at the different Gruen regions (Bodén et al. 2004).

### Statistics

The Mann-Whitney U test for non-parametric variables (independent groups) was used to compare Charnley's functional class (A and C) with HHS, and to compare bone defects prior to revision with subsequent bone loss. The Wilcoxon signed-rank test (paired observations) was used to compare side differences in BMD. The statistical analyses were performed with the statistical package JMP (SAS Institute, Cary, NC, USA). Differences were considered statistically significant at p-values < 0.05.

## Results

### Clinical results

The mean HHS for all patients was 74 (30–100) points. We noted a lower HHS among patients in Charnley's function class C (mean 58 points) compared to patients in class A (mean 86 points) (p = 0.003). 3 patients had moderate thigh pain and 1 patient had mild pain. 9 patients limped, 10 used walking support but 3 of these patients used a cane for long walks only.

### Radiographic results

**Figure 2.**
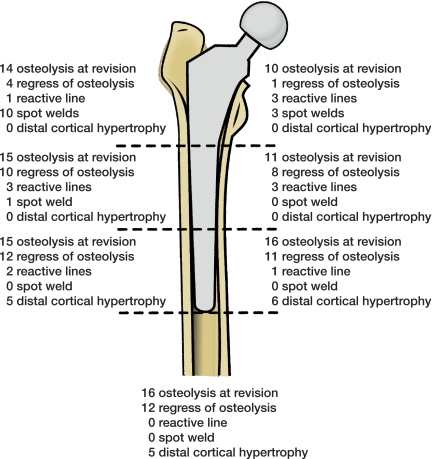
Radiographic findings in the different Gruen regions. Linear osteolysis at revision and remodeling after the rearthroplasty.

We saw no stem loosening after the rearthroplasty. Linear osteolysis at revision was evenly distributed in all regions and regression of osteolysis was more commonly noted in the distal regions (Table 2). Signs of stability (endosteal bone bridges i.e. spot welds, absence of radio-opaque lines in the coated area, and absence of radiolucent lines along the stem) were found proximally in half of the hips, and cortical hypertrophy was seen distally in one quarter of the femurs. Two stems had subsided > 4 mm at follow-up; however, they showed other signs of stability (spot welds and absence of radiolucent lines along the stem). The maximum subsidence was 7 mm. None of the stems migrated into varus or valgus. Several signs of remodeling were observed (Figure 2). We also examined the acetabular components, although they were not the focus of this study. There were no loose cups, defined as absence of circumferent radiolucency, migration, or reorientation of the cup.

**Table 2.** Bone remodeling at follow-up (22 hips)

Stem stability parameters	
Fixated stems	22
Subsidence (maximum 7 mm)	2
Change in varus-valgus alignment	0
Spot welds	12
Stress-shielding parameters	
Calcar resorption	8
Calcar “round-off”	2
Distal cortical hypertrophy (1–4 mm)	6
Bone remodeling parameters	
Osteolysis at revision	21
Regression of osteolysis	19
Newly formed osteolysis	1
Unspecific parameters	
Pedestal formation	16
Reactive lines	7
Heterotopic ossification	
grade 1	6
grade 2	2
grade 3	1
grade 4	0

### Bone mineral changes

**Figure 3.**
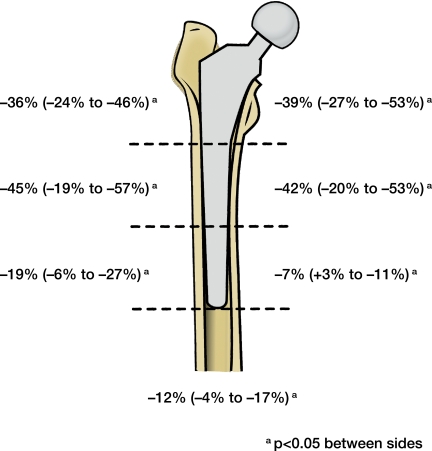
Percentage side difference in BMD in different Gruen regions after the rearthroplasty. Median values (25–75 percentiles) are given. An asterisk indicates a difference between sides (p < 0.05).

We found a marked reduction in BMD on the reoperated femur in all regions compared to the unoperated side (p < 0.001 for all regions) (Figure 3); the largest reduction was noted in regions 1–2 and 6–7 where it was reduced by 36–45%. We found no difference in BMD loss due to grade of bone defects prior to revision (Gustilo type I–II/Endo-Klinik type II–III). The patients who underwent bone grafting around the proximal part of the stem at revision had the same amount of bone loss as the patients who were not grafted.

### Complications

6 hips had dislocated, 3 of them more than once. 1 recurrent dislocation was successfully treated with exchange of liner and a larger head size. Another recurrent dislocation was treated with an orthosis for a few weeks after the last closed reduction and no dislocations occurred thereafter. The sixth patient with an unstable hip (recurrent) had cerebral palsy and has undergone 6 closed reductions with no dislocations in recent years. 1 trochanteric fracture occurred 6 months after revision; the fracture was diagnosed late and it was treated conservatively with a good result. 1 patient had a postoperative wound infection and 1 patient got a clinically apparent deep vein thrombosis. No case of pulmonary embolus, neurological or vascular complications was seen.

## Discussion

The results after cemented revision arthroplasty are not encouraging (Mulroy and Harris 1996, Strömberg and Herberts 1996, Haydon et al. 2004). However, a recent study by Howie et al. (2007) showed excellent results after cemented revision using a collarless double-tapered stem. In order to reduce loosening, different uncemented prosthetic designs have been introduced; most of the studies have used extensively coated femoral implants or distally anchored stems (Lawrence et al. 1994, Paprosky et al. 1999, Moreland and Moreno 2001). They show good results both clinically and radiographically. However, there could be a disadvantage with an extensively fixated implant. The distal fixation, and the fact that the implant is stiffer than the surrounding bone, inevitably results in unloading of the proximal bone. According to Wolff's law, this would give a reduced bone stock proximally. This phenomenon might be a problem in the future: theoretically, there is a risk of fractures or avulsions of muscle insertions in the trochanteric region. Proximally coated—and therefore proximally fixated—uncemented femoral implants used in hip revision may have the advantage of minimizing further bone loss proximally.

In this paper, we report the bone loss around 22 implants after rearthroplasty with an uncemented hydroxyapatite-coated prosthesis due to aseptic loosening at a mean of 6 years. We have used this prosthesis in cases where the bone stock could give direct rigid stability for the implant, i.e. in revisions where the bone deficiencies were mild to moderate. Clinically, the patients in this study did well after the revision, achieving a mean HHS of 74 points at follow-up.

### Radiographic findings

In our series, only 2 femoral components subsided but they also showed several radiographic signs of stable fixation. We saw few reactive lines and more than half of the femurs showed formation of endosteal bone bridging (spot welds); these are considered to be associated with good fixation and osseointegration of uncemented stems (Engh et al. 1990).

Stress-shielding is a well-known phenomenon around uncemented stems. The Bi-Metric stem is designed to reduce the stress-shielding effect by a porous coating aimed at osseointegration only in the proximal region. However, our radiographic data revealed that this is not the case. We saw several signs of stress-shielding, such as calcar resorption (8 hips) and distal cortical hypertrophy (6 hips). Distal cortical hypertrophy and proximal resorption are consequences of stress transfer; the frequencies are in accordance with previously reported remodeling around the same stem in primary arthroplasty at 5 years postoperatively (Bodén et al. 2006a). Linear osteolysis, present at revision in all hips but 1, filled out with new bone formation onto the uncemented stem in 19 of the cases. Despite this, we found a large loss of BMD.

Bone mineral measurement after an uncemented re-arthroplasty is an important means of investigating the reaction upon the bone after implantation of the stem. Of particular interest is the determination of whether new bone is forming or whether pre-existing bone is being resorbed in response to the altered patterns of stress that are created by the components. The use of an uncemented prosthesis in revision surgery also presents the opportunity for restoration of lost bone stock with grafting (Engh et al. 1988). Bone was grafted around the proximal part of the stem in more than half of our operations. Despite this, we noted a profound loss of bone in the proximal regions. Most studies are done by visual inspection and comparison of film images that offer a rather insensitive measurement of bone changes. DEXA provides a more sensitive assessment of bone changes around prosthetic implants and has been shown to be a safe (Kalender 1992), accurate (Kröger et al. 1996), and reproducible method of measuring periprosthetic bone remodeling. With this technique, changes may be seen as early as 6 months after the operation (Cohen and Rushton 1995, Bodén and Adolphson 2004).

A large loss of BMD in the proximal femur has been reported by many authors in longitudinal studies following primary uncemented arthroplasty (Marchetti et al. 1996, Nishii et al. 1997, Rosenthall et al. 1999, Venesmaa et al. 2001). In the calcar area, where maximal bone resorption is expected, the bone loss in these studies has varied between 16% and 30%, depending on the study design and the implant used. In a previous study of the Bi-Metric stem with DEXA after primary arthroplasty, we found bone loss of around 30% in regions 1 and 7 after 6 years but very small changes in other regions (Bodén et al. 2006b). In the present study, in the reoperated femur, we found a marked reduction in BMD in all regions, the largest reduction being noted in regions 1, 2, 6, and 7 (36–45%). Osteolysis, present at revision in all hips but 1, may contribute to a lower periprosthetic BMD. However, the sites with linear and focal osteolysis at revision had been filled out with new bone onto the uncemented stem in 19 of our cases. Despite this, we found a large loss of BMD, indicating that the new bone formation is less dense in quality. The fact that we did not find any correlation between degree of osteolysis at revision and subsequent loss of BMD also points to other causes such as pre- and peroperative changes and inactivity-related bone resorption, factors that a cross-sectional study like this cannot assess adequately.

The use of an uncemented prosthesis in revision surgery presents the opportunity for restoration of lost bone stock with grafting (Engh et al. 1988). In another study of BMD changes after uncemented rearthroplasty, Nesse et al. (2003) presented a material where all patients received impacted bone allograft. They noted less proximal bone loss than we did, which may be due to the fact that we grafted only half of the patients.

There are some limitations to our investigation. It is a cross-sectional study, where periprosthetic BMD is compared with that on the healthy side. This could be a cause of error, because of a possible side difference in BMD before revision. Thus, the BMD difference found after rearthroplasty in cross-sectional studies may be overestimated and only a longitudinal study would give more accurate information about the remodeling process. In retrospective studies, pre- and peroperative BMD have been ignored because preoperative BMD of the involved hip is not available for comparison. Reduced activity of the affected leg, both pre- and postoperatively, can contribute to differences (Marchetti et al. 1996)—as can the surgical procedures and the hip pathology itself (Kröger et al. 1996, Martini et al. 2000). In a cross-sectional study, it is important to perform the investigation after the changes have stabilized. Hughes et al. (1995) considered that approximately 3 years (after which time most remodeling was complete) is an optimum time to assess atrophy of the proximal femur.

In summary, we found good medium-term clinical and radiographic results with the uncemented Bi-Metric femoral stem prosthesis in revision. Compared to primary THA, a similar remodeling pattern was seen but a larger and more widespread degree of bone loss in the proximal femur was noted with DEXA after rearthroplasty. Long-term results will show whether this could lead to mechanical complications such as loosening or fractures.
